# *Haemophilus influenzae* Type b Invasive Disease in Amish Children, Missouri, USA, 2014

**DOI:** 10.3201/eid2301.160593

**Published:** 2017-01

**Authors:** Angela L. Myers, Mary Anne Jackson, Lixin Zhang, Douglas S. Swanson, Janet R. Gilsdorf

**Affiliations:** Children’s Mercy Hospital, Kansas City, Missouri, USA (A.L. Myers, M.A. Jackson, D.S. Swanson);; Michigan State University, East Lansing, Michigan, USA (L. Zhang);; University of Michigan Medical Center, Ann Arbor, Michigan (J.R. Gilsdorf)

**Keywords:** Haemophilus influenzae type b, Hib, invasive disease, bacteria, Amish, United States, Missouri, children, epidemiology, at risk, transmission, unvaccinated carriers, unvaccinated, underimmunized communities, undervaccinated, undervaccinated communities, vaccine hesitancy, vaccines

## Abstract

During 5 months in 2014, three Amish children in Missouri, USA, were diagnosed with invasive *Haemophilus influenzae* type b infection. Two were rural neighbors infected with a genetically similar rare strain, sequence type 45. One child had recently traveled, raising the possibility of maintenance of this strain among unvaccinated carriers in Amish communities.

*Haemophilus influenzae* type b (Hib) vaccine was introduced in the United States in 1985, and since then, the incidence of invasive Hib infection among young children (<5 years of age) has decreased by 99%, from 46–100 cases/100,000 children to <1 case/100,000 children (*1*–*4*). However, small pockets of unimmunized and underimmunized children remain in this country and may continue to serve as potential reservoirs for disease. In a cluster of cases that occurred during 1999–2000 among Amish children in Pennsylvania, USA, 3 of 8 strains were genetically similar and identified by multilocus sequence typing (MLST) as sequence type (ST) 45, a previously unreported strain type in the United States (*5*). In 2014, we confirmed invasive Hib disease in 3 Amish children from 2 different communities in Missouri; 2 patients had disease caused by the ST45 strain that was implicated in the 1999–2000 Hib cluster in Pennsylvania (Table).

**Table:** Demographic and infection and characteristics for 3 Amish children infected with *Haemophilus influenzae* type b strains, Missouri, USA, 2014*

Patient no., community	Patient age, mo	Month of Hib diagnosis	Infection	Outcome	Capsule genes *bexA/bexB*	*sodC*	MLST
1, A	13	Jan	Septic arthritis	Recovered	+/+	−	ST6
2, B	24	Feb	Epiglottitis	Died	+/+	−	ST45
3, B	13	May	Septic arthritis	Recovered	+/+	−	ST45

*None of the children had previous underlying conditions. *bexA*, b capsule expression A; *bexB*, b capsule expression B; community A, Amish community in southwestern Missouri; community B, Amish community in northwestern Missouri; MLST, multilocus sequence type; *sodC,* superoxide dismutase gene.

## The Patients

Patient 1 was an unimmunized 13-month-old Amish boy from southwestern Missouri (community A) who had a fever (40°C) and refused to bear weight on his left leg. Blood and synovial fluid grew β-lactamase–negative Hib. After receiving parenteral ceftriaxone for 10 days, the patient was transitioned to oral amoxicillin to complete a 21-day course of antimicrobial therapy. At follow-up 2 weeks after hospital discharge, all signs and symptoms of infection had resolved.

Patient 2 was an unimmunized 2-year-old Amish girl from rural northwestern Missouri (community B), 250 miles north of community A. She had a fever (39°C) and throat pain with drooling and difficulty breathing. She required immediate intubation for epiglottitis, and vancomycin and ceftriaxone were initiated. Blood cultures grew Hib, and a tracheal culture grew methicillin-susceptible *Staphylococcus aureus* and Hib. She suffered severe neurologic injury, and care was withdrawn per parent request. The patient subsequently died.

Patient 3 was an unimmunized 13-month-old Amish girl from northwestern Missouri (community B) and a neighbor of patient 2. She had a history of a fall from a wagon 4 weeks before hospital admission. She had persistent pain and limited range of motion of the right leg, which had become swollen 2 weeks before hospitalization. Magnetic resonance imaging at admission showed osteomyelitis of the right acetabulum, with dislocation of the right femoral head with necrosis, and extensive soft tissue and muscular abscesses around the proximal femur and into the right pelvis and lower abdominal retroperitoneum. Synovial fluid culture grew Hib. The patient underwent 3 operative washout procedures and placement of a spica cast. After 10 days of intravenous therapy with cefepime followed by ampicillin, she was transitioned to oral amoxicillin to complete a total of 6 weeks of therapy, after which she was fully recovered.

The Missouri State Health Department performed confirmatory serologic testing on all isolates, using antiserum for Hib capsular types a–f (Becton, Dickinson and Company, Franklin Lakes, NJ, USA). We used PCR, as previously described (*6*,*7*), to test all *H. influenzae* strains in this cluster for specific capsule types a–f. We also used PCR to test the strains for capsule genes *bexA* and *bexB* (b capsule expression A and b capsule expression B) and for superoxide dismutase gene (*sodC*) to distinguish division I (*sodC*−) from division II (*sodC*+) *H. influenzae* (*8*)*.* We performed MLST using the 7 standard MLST alleles. Whole-genome sequencing of all 3 *H. influenzae* strains was performed by Illumina (http://www.illumina.com/techniques/sequencing.html); the strain from patient 1 was sequenced a second time by PacBio (http://www.pacb.com/) (*9*,*10*). The 2 children from community B were infected with nearly identical Hib strains identified as ST45 (Technical Appendix Figure); the child from community A was infected with a genetically different strain, ST6. The ST45 Hib strain is rarely reported and represents only 3 (0.5%) of 598 type b strains and 3 (0.15%) of 1,982 *H. influenzae* strains in the MLST database (*10*).

During December 1999–February 2000 in Pennsylvania, 7 cases of Hib infection were identified in children <3 years of age (*3*). Six of these cases occurred in Amish communities (*5*). Among the 7 Hib isolates, 2 were ST6 strains from 2 different communities, 3 were ST45 strains from 3 different communities, and 2 were ST44 strains (community source not available). We are not aware of subsequent ST45-related cases until now.

Although we found no epidemiologic link between the 2 Missouri children infected with Hib ST45 strains and Amish communities in Pennsylvania, patient 2 had traveled to Indiana and Wisconsin to visit family before the infection developed, raising the possibility of contact with a carrier of this rare strain among unvaccinated children in Amish communities. To enable evaluation of possible epidemiologic links, strain sequence typing should be considered for cases of invasive Hib disease, especially in the setting of underimmunized communities.

## Conclusions

Since the implementation of Hib conjugate vaccination, the incidence of Hib disease in the United States has markedly declined. By reducing asymptomatic nasopharyngeal carriage, high rates of vaccination provide herd immunity protection for undervaccinated children. However, in underimmunized communities, relatively high prevalence rates of Hib carriage can serve as a reservoir for the organism (*3*), and our report illustrates that children from underimmunized communities remain at risk for serious Hib disease. Although now uncommon in the United States, Hib disease must be considered in the differential diagnosis of unimmunized and undervaccinated children with symptoms compatible with Hib infection.

Immunization is not forbidden by Amish religious teachings, but vaccination rates are generally low in many Amish communities (*3*,*11*). Surveys of Amish communities have identified fear of side effects, philosophical objections, and lack of priority as some reasons for vaccine hesitancy (*3*,*11*). Parents of one of our patients reported worry about vaccine side effects and preference for more natural healthcare as reasons for not immunizing their children. Thoughtful, respectfully delivered public health education may help influence Amish parents to accept vaccines (*3*,*11*). 

Although there was no known contact between patients 2 and 3, the local health department initiated a vaccination campaign within community B because the 2 Hib cases occurred within a 3-month period. Over the course of a year after these 2 cases in community B, the local health department provided vaccine to children <5 years of age on 4 separate occasions. Of the vaccine-eligible children (n = 40), 35 had completed the series as of July 2015, and the other 5 were progressing toward completion. A mass vaccination campaign was not undertaken in community A. However, the patient’s siblings ultimately received Hib vaccine. Familiarity with the recent cases and education about Hib disease and vaccines likely influenced the generally successful Hib vaccine campaign in community B. Efforts to identify and appreciate obstacles to vaccine utilization among Amish and other undervaccinated communities aid health departments and clinicians in their efforts to improve community education and prevent infection.

Technical Appendix eBURST depiction of *Haemophilus influenzae* type b strains within the MLST database.
