# Supplementary material for: Haemophilus influenzae Type b Invasive Disease in Amish Children, Missouri, USA, 2014
Source: Emerg Infect Dis. 2017 Jan;23(1):112–4. doi: 10.3201/eid2301.160593 (PMC5176238; doi:10.3201/eid2301.160593)
Supplement: Technical Appendix — eBURST depiction of Haemophilus influenzae type b strains within the MLST database. [file 16-0593-Techapp-s1.pdf]

Article DOI: http://dx.doi.org/10.3201/eid2301.160593

Haemophilus influenzae Type b Invasive Disease in Amish Children, Missouri, USA, 2014

Technical Appendix

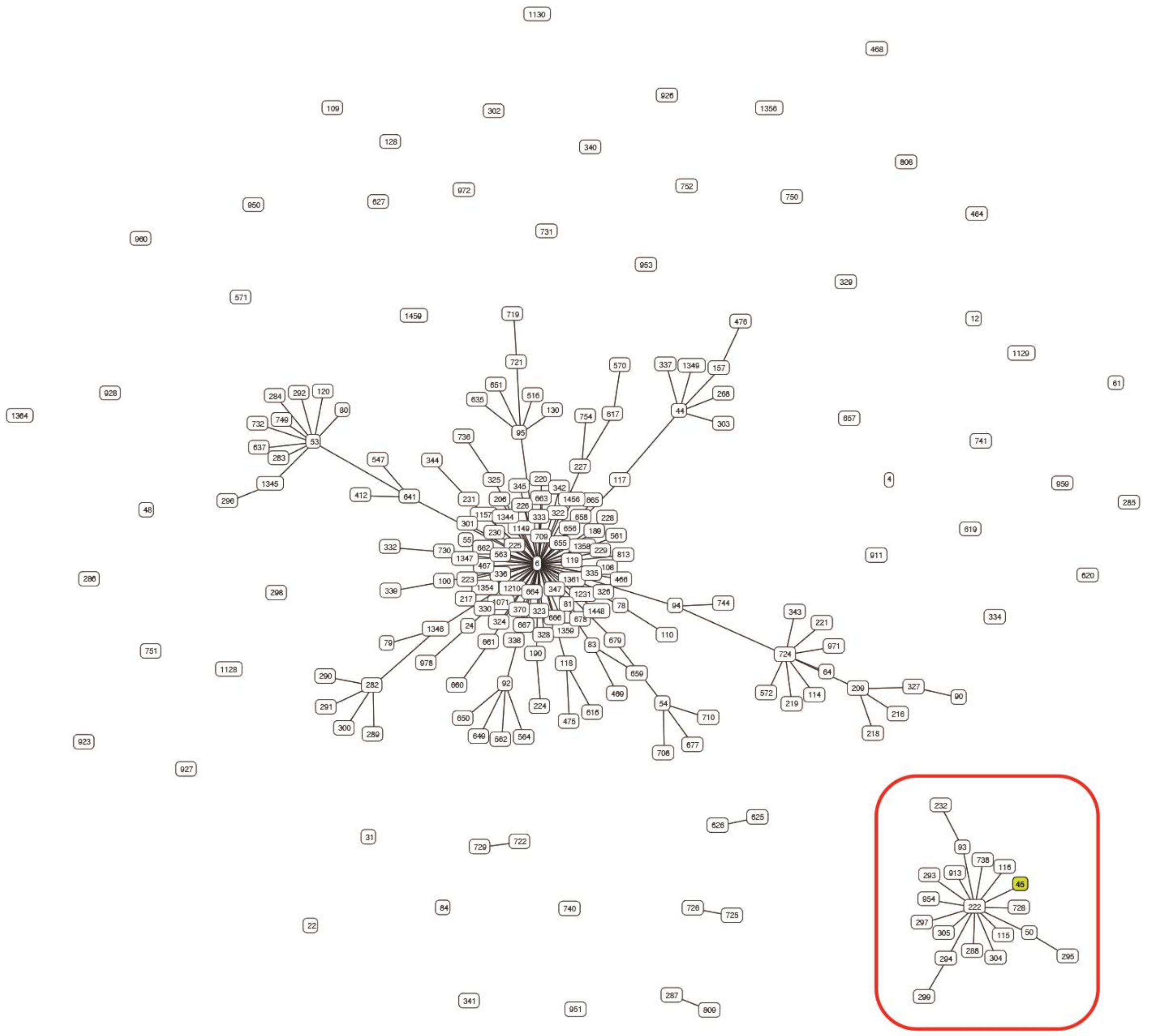

Technical Appendix Figure. eBURST depiction of *Haemophilus influenzae* type b strains within the MLST database (*1*). The center of the large BURST diagram indicates ST6 strains; yellow box in the small BURST diagram indicates ST45 strains. Strains linked by a line differ by at least 1 nucleotide in 1 of the 7 multilocus sequence typing alleles. ST, sequence type.

Reference

1. PubMLST. *Haemophilus influenzae* MLST Databases [cited 2015 Jul 20]. http://pubmlst.org/hinfluenzae/</eref>
